# Incorporating Biomarker Stratification into STAMPEDE: an Adaptive Multi-arm, Multi-stage Trial Platform

**DOI:** 10.1016/j.clon.2017.10.004

**Published:** 2017-12

**Authors:** C. Gilson, S. Chowdhury, M.K.B. Parmar, M.R. Sydes

**Affiliations:** ∗ MRC Clinical Trials Unit at UCL, London, UK; † Guy's and St Thomas' NHS Foundation Trust, London, UK

**Keywords:** Biomarkers, clinical trial design, platform trials, precision medicine, prostate cancer

## Abstract

The treatment and outcomes for advanced prostate cancer have experienced significant progress over recent years. Importantly, the additional benefits of ‘up front’ chemotherapy (docetaxel) and abiraterone, over and above conventional androgen deprivation, have been separately demonstrated in the multi-arm, multi-stage (MAMS) STAMPEDE protocol, which continues recruitment to other questions. Alongside this, insights into the underlying molecular biology and, inevitably, the molecular heterogeneity of prostate cancer are opening the door to new therapeutic approaches. Incorporating this understanding and testing these hypotheses within STAMPEDE brings new challenges to the MAMS approach, but has the potential to further improve the outlook for this disease.

## Statement of Search Strategies Used and Sources of Information

This overview reflects the opinion and experience of the authors and evidence has been presented accordingly. It is based upon our own research findings and clinical trial experience. It is not a systematic review.

## Introduction

The last decade has seen major advances in both the understanding and the treatment of advanced prostate cancer, with a number of agents gaining approval as standard of care (SOC) in castrate-resistant disease. More recently, the additional benefit of using these drugs earlier, at first presentation, has also been shown and is now considered SOC [1], [2], [3], [4]. In parallel, there has been significant progress in understanding prostate cancer biology that promises to further improve outcomes, but by nature moves away from a ‘one size fits all’ approach. Implementing precision medicine requires the validation of putative predictive biomarkers within well-designed clinical trials. Analysis of representative samples obtained as part of a clinical trial protocol can support many stages of biomarker discovery, assay development and qualification, as laid out in the Cancer Research UK Biomarker Roadmap [5]. Knowledge gained through the genomic characterisation of advanced metastatic prostate cancer provides both the rationale and the means to progress this research priority in the first-line setting.

It has been shown that around 20% of metastatic castrate-resistant prostate cancers (mCRPC) have loss-of-function somatic genomic aberrations or germline deficiencies in genes involved in DNA repair, in particular those involved in the repair of double-stranded DNA breaks using homologous recombination. The resulting homologous recombination deficiency (HRD) supports synthetic lethality as a therapeutic approach in prostate cancer. Importantly, the poly(ADP-ribose) polymerase (PARP) inhibitor, olaparib, has been shown to benefit this group, providing the proof-of-concept for this precision approach [6], [7].

However, the greatest absolute benefit of effective therapies may be observed when used early, at the initiation of long-term androgen-deprivation therapy (ADT) [1], [2], [3], [4]. Furthermore, prostate cancers exhibit significant intratumoural genetic heterogeneity, which increases in advanced disease in response to multiple lines of therapy [8], [9]. Mechanistically, DNA repair-deficient cancers can be expected to acquire and tolerate somatic mutations at a greater rate, which may thwart targeted precision medicine approaches due to the increasing likelihood of acquired secondary resistance and risk of sampling bias due to the spatial genetic heterogeneity observed in metastatic prostate cancer [10], [11]. Together, this provides the rationale to evaluate precision medicine approaches earlier, with the aim of achieving the greatest impact on patient outcome.

Approval from Cancer Research UK and independent scientific peer-review has been obtained to evaluate rucaparib within the STAMPEDE trial platform. Here we set out the considerations and challenges faced when incorporating biomarker stratification within an adaptive trial platform, which will be the first example of a biomarker-directed treatment strategy in this disease setting.

## STAMPEDE

**Fig 1:**
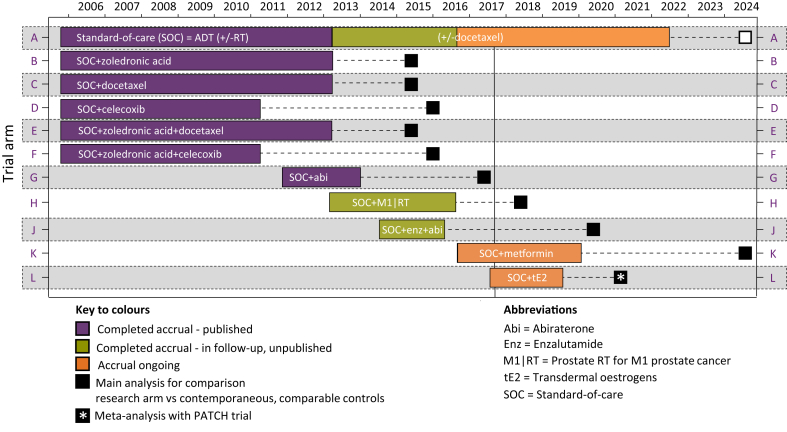
Arms of the STAMPEDE trial platform over time.

STAMPEDE (Systemic Therapy in Advancing or Metastatic Prostate Cancer: Evaluation of Drug Efficacy) is a well-established randomised controlled trial that recruits men with high-risk locally advanced or metastatic prostate cancer who are commencing long-term ADT for the first time, termed hormone-naive prostate cancer (HNPC) [12]. The trial uses a multi-arm multi-stage (MAMS) platform design: multi-arm because many treatment approaches can be tested simultaneously; multi-stage because pre-specified interim analyses can be used to stop recruitment early to arms showing insufficient evidence of activity [13]. Data from all stages are included in the final analysis of efficacy, powered on the primary outcome, overall survival. The trial opened in 2005 with five ‘original comparisons’ evaluating the efficacy of adding docetaxel, zoledronic acid and celecoxib, given alone or in combination, to the then SOC, ADT ± prostate radiotherapy. Since then a number of new research arms have been added to undertake randomised comparisons of: abiraterone; prostate radiotherapy for patients with newly diagnosed metastatic disease (M1|RT); enzalutamide given in combination with abiraterone; metformin, an anti-diabetic medication; and, most recently, transdermal oestradiol, a proposed alternative form of ADT (see Figure 1).

## Comparisons: Reported, Unreported and Ongoing

The results of the ‘original comparisons’ and the ‘abiraterone comparison’ have been reported, with both docetaxel and abiraterone shown to significantly improve survival [2], [3]. The addition of docetaxel improved median survival from 71 months to 81 months; hazard ratio 0.78 (95% confidence interval 0.66–0.93); *P* = 0.006. The strength of evidence is most clearly apparent within the metastatic subgroup where the benefit is also reflected in the results of the CHAARTED trial. Both trials contributed to the STOpCaP meta-analysis; aggregate data from 3206 patients with metastatic HNPC showed that docetaxel improved 4 year survival by 9% (hazard ratio 0.77; 95% confidence interval 0.68–0.87; *P* < 0.0001) [14]. These data have been practice changing [15]. The results of the ‘abiraterone comparison’ also show improved outcome; 3 year survival improved from 76% to 83%; hazard ratio 0.63 (95% confidence interval 0.52–0.76) [3]. Opportunistic data acquired through the overlapping randomised population accrued between 2011 and 2014 were presented at ESMO 2017 and suggest superior progression-free survival with abiraterone but a comparable survival outcome [16]. The ‘M1|RT comparison’ and ‘enzalutamide and abiraterone comparison’ continue in follow-up, with survival results expected in the next 1–3 years. Recruitment is ongoing to two comparisons evaluating metformin and transdermal oestradiol as re-purposed anti-cancer therapies, both proposed to mitigate the adverse cardiovascular and metabolic effects of long-term androgen suppression [17], [18]. STAMPEDE is investigating whether adding metformin to the current SOC for non-diabetic men can improve all-cause survival, and whether transdermal oestradiol, shown to offer superior cardiovascular, quality-of-life and bone health outcome, is non-inferior based on survival [19], [20], [21].

## Rationale to Incorporate Biomarker Selection within STAMPEDE

**Fig 2:**
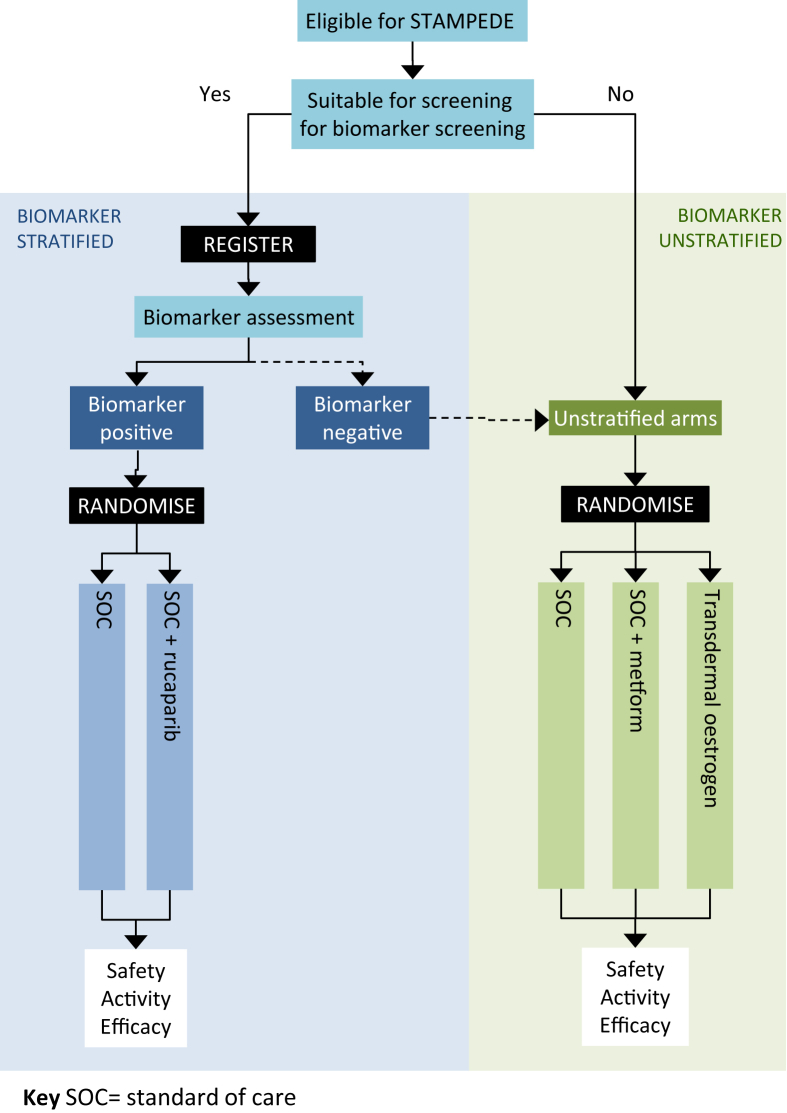
Future STAMPEDE randomisation schema.

The ‘rucaparib comparison’ will be embedded into STAMPEDE by way of a protocol amendment as the most efficient route to address this question in this disease setting (see Figure 2). Through further adaptation, we avoid establishing a competing biomarker-selected trial within an overlapping population, which could risk impacting on accrual and potentially generalisability of results through depletion of biomarker-defined groups hypothesised to have both prognostic and predictive effects. Through incorporation we can determine and control the impact on the ongoing comparisons and continue to answer important research questions for both biomarker-positive and -negative patients through an inclusive trial platform. This is key to sustaining efficient accrual to all comparisons and conducting cost-effective evaluation of new agents, particularly for those targeted at low-frequency biomarkers.

## Biomarker Stratification: Considerations

When planning biomarker-enriched clinical trials requiring prospective biomarker characterisation there are several aspects that require consideration to inform the trial design and feasibility of implementation (summarised in Table 1).

**Table 1:** Considerations for biomarker-enriched trial designs

Framework for incorporating biomarker stratification in a platform trial
1. Can the biomarker of interest be reliably measured using a validated assay?
2. What is test-performance in clinically available samples representative of the population of interest?
3. Is the biomarker prognostic necessitating a separate control in order to distinguish a prognostic from a predictive effect?
4. What is the biomarker prevalence in the population of interest?
5. What is the strength of evidence of a predictive effect, i.e. the specificity of the biomarker?
6. What is the strength of evidence to support the rationale and clinical efficacy of the targeted therapy in the biomarker-defined group?
7. What is the overlap between this biomarker-defined group and others of interest?
8. What are the implications for other overlapping accruing comparisons?

### Biomarker Measurement

The reliable identification of the biomarker-positive population of interest requires an analytically validated assay shown to perform to an acceptable standard in clinically available samples [22]. In the case of the STAMPEDE trial population, these are typically small, prostate core biopsies stored as formalin-fixed, paraffin-embedded tumour blocks. Preliminary data have highlighted the impact of pre-analytical variables such as variation between sites' fixation protocols in the ability to extract sufficiently high-quality DNA required for analysis. This has been recognised by others; Genomic England have reported that the quality of routinely obtained cancer samples may often be suboptimal for molecular analysis and are leading several initiatives aiming to improve sample quality and inform the most suitable methods for sample collection and storage [23]. The clinical utility of the method of biomarker measurement should be assessed in representative clinical samples to estimate the test-failure rate. This in turn informs the numbers needed to screen, a crucial parameter in defining the cost, accrual time and, therefore, feasibility of implementation. The impact of a high test failure rate is greatest when aiming to evaluate low-prevalence biomarkers when this can render a trial infeasible.

To be workable within a clinical trial screening window the biomarker assessment must be available within a reasonable timeframe. Several factors will affect turnaround around times, including pathology department resources, shipping distances, the capacity of the biomarker provider and the need to batch for cost-efficiency. The risk of a long turnaround time is that randomisation and, therefore, treatment, is delayed. The effect of this will probably be greatest in trials recruiting patients with progressive disease where a participant's clinical status may change, making them ineligible during the time taken to complete screening. However, in all disease settings, the time required to undertake biomarker analysis should not risk disadvantaging patients' access to treatment. In trial platforms where multiple randomisations are possible, biomarker analysis should be completed in such time that patients remain eligible for all possible randomisations. This is relevant for STAMPEDE, which will continue to randomise to non-biomarker selected comparisons.

### Prognostic Importance

Aberrations in BRCA and other HRD genes are negatively prognostic in prostate cancer and, therefore, patients allocated to the research arm of the ‘rucaparib comparison’ will only be evaluated against comparable biomarker-positive controls [24], [25], [26], [27]. Evidence of a prognostic effect can inform the design of biomarker-enriched trials and affect the assessment of feasibility, particularly of low-frequency biomarkers. When considering a biomarker-selected randomisation within a platform trial, knowledge of a prognostic effect informs whether it is justifiable to share a control arm, with resulting efficiencies (namely fewer control arm patients). If the biomarker is known to be prognostic, separate comparisons are required in order to distinguish a predictive from a prognostic effect, as exemplified by the design of FOCUS-4 [28]. Knowledge of a prognostic effect can also inform the size of a trial powered to detect a difference in time-to-event outcomes. If the survival time is shorter, the information required for reliable analyses (e.g. number of deaths for a trial powered on the primary outcome of survival) will be accrued sooner and, therefore, a smaller trial may be required.

However, the acquisition of robust prognostic information for emerging biomarker-defined groups can be challenging. Retrospective retrieval of archival samples is vulnerable to bias, with the risk that those patients who have subsequently progressed and enrolled on mCPRC trials requiring archival tissue analysis are under-represented. Additionally, it has been suggested that the quality of the DNA extracted from formalin-fixed paraffin-embedded samples declines with sample age; this has been supported by the experience of the STAMPEDE group to date. It is hoped that through data sharing, for example through contribution to genetic consortia, it will be possible to inform clinical trial design and target research efforts in poor prognostic genetic subpopulations, aiming to improve outcome in those patients with the greatest unmet clinical need.

### Biomarker Prevalence

The duration of recruitment to the ‘rucaparib comparison’ will depend on the frequency of DNA repair defects in men eligible for STAMPEDE. Evidence from mCRPC cohorts and primary localised prostate cancer has that shown DNA repair defects are more common in metastatic disease [8], [29], [30], [31], [32]. Around 20% of mCRPC cohorts (range 7–27%) have detectable mutations in one or more of 14 genes involved in homologous recombination, including BRCA1, BRCA2, ATM, BARD1, CHEK2, PALB2, RAD51 [6], [7], [9], [33], [34], [35], [36], [37], [38], [39], [40]. The vast majority of the prostate cancer cohorts profiled to date have either consisted of men with advanced, heavily pre-treated CRPC or localised prostate cancer suitable for radical prostatectomy. Therefore, knowledge of the genomic landscape of men presenting with high-risk locally advanced or metastatic prostate cancer (i.e. eligible for STAMPEDE) is currently very limited. Furthermore, all sequenced mCRPC series to date involve patients participating in trials, precision medicine initiatives or autopsy programmes at tertiary academic centres and are, therefore, vulnerable to selection bias (see Table 2) [6], [7], [9], [34], [36], [37].

**Table 2:** DNA repair deficiency in prostate cancer: summary of prevalence data

Ref	Cohort details	M0 (n)	M1 (n)	% BRCAm	% HRD*
[30]	HNPC suitable for prostatectomy	112	-	1%	4%
[31]	Low/intermediate risk HNPC	333	-	4%	13%
[29]	Mixed HNPC	55	2	0%	11%
[32]	Mixed cohort, predominantly M0	181	37	0	0
[8]	Mixed cohort, both HNPC and mCRPC	25	20	12%	20%
[29]	Mixed, fatal mCRPC sampled at rapid autopsy and HNPC suitable for prostatectomy	11	50	2%	7%
[34]	Selected due to unusual clinical course, suspected predisposition, e.g. family history or atypical histology	29	13	16% (10% gBRCA)	27% (24% gHRD)
[36]	Fatal mCPRC sampled at rapid autopsy		54	7%	16%
[6]	mCRPC trial participants at academic centres	-	150	14%	23%
[7]	mCRPC in an unselected PARPi trial	-	50	14%	27%
[37]	Cohorts participating in clinical trials, rapid autopsy programmes or precision medicine initiatives at academic centres		692	6.2% (gBRCA)	11.2% (gHRD)
[35]	Sporadic mCRPC eligible for abiraterone +/- PARPi	-	80		25%
[33]	Sporadic mCRPC eligible for PROREPAIR-B (prospective cohort study)	-	419	4.2% (gBRCA)	9.1% (gHRD)

BRCAm = BRCA mutant, CNA = copy number alteration, gBRCA = germline BRCA mutation, gHRD = germline HRD mutation, HNPC = hormone-naïve prostate cancer, M0 = non-metastatic prostate cancer, M1 = metastatic prostate cancer, tNGS = targeted next generation sequencing, mCRPC = metastatic castrate resistant prostate cancer, PARPi = PARP inhibitor, WES = whole exome sequencing.

* Homologous recombination deficiency defined as pathogenic aberration in one or more: BRCA1, BRCA2, ATM, BARD1, BRIP, CDK12, CHEK2, NBN, PALB2, RAD51, RAD51B, RAD51C, RAD51D, RAD54L.

Eligibility to the ‘rucaparib comparison’ will be limited to metastatic HNPC with a detectable pathogenic mutation in one or more of 14 HRD-related genes and the trial design has been developed based on an estimated biomarker prevalence of 10–15%. A feasibility assessment is planned 1 year after the activation of recruitment; accumulating prevalence data acquired in the screened population will be reviewed and adjustments to the target screening accrual numbers made accordingly.

The frequency of the biomarker of interest in the target population is crucial in developing the best approach to therapeutic evaluation within a biomarker-enriched trial. Trials restricting enrolment to low-prevalent biomarker groups risk being very costly, given the numbers needed to screen; additionally, the high screen failure rates can deter patients and investigators, negatively impacting on accrual. This contributes to the rationale for incorporating both biomarker-selected and -unselected randomisations within one trial platform, in order to sustain accrual to low-prevalence biomarker groups.

### Evidence of a Predictive Effect

Eligibility for the ‘rucaparib comparison’ will be restricted to the biomarker-positive population based on the evidence of a predictive effect demonstrated for PARP inhibition in mCRPC. Justification of biomarker enrichment requires evaluating the strength of evidence of a predictive effect; this also informs the effect size [41]. In the scenario that the evidence of a predictive effect is judged insufficient to limit treatment to only biomarker-positive patients, it may be preferable to enrol an unselected population and, through prospective biomarker assessment, stratify by biomarker status to enable pre-planned subgroup analyses. If, however, there is good evidence of a predictive effect, such that enrichment can be justified, randomisation may be limited to the biomarker population. One approach to the challenge of defining the level of evidence required is to adopt a pragmatic approach: would randomisation of an unselected population be acceptable and, therefore, feasible? Here, evaluation within an adaptive MAMS trial platform is advantageous as it is possible to initially randomise the population with the strongest evidence of a predictive effect and then subsequently activate randomisation in a broader group should sufficient activity be shown. Early stopping rules could be used in this scenario, but the value of being able to test the specificity of the marker is high: it may be that the research treatment could offer a broader benefit, which risks being missed if an enrichment design is adopted and not subsequently evaluated.

The magnitude of the targeted treatment effect is also dependent on the evidence of a predictive effect; where there is strong evidence of a predictive effect in a biomarker-defined group, the treatment effect may be expected to be greater than for a non-targeted therapy in an unselected population. The vast majority of data demonstrating the efficacy of PARP inhibition have been acquired in the setting of ovarian or breast cancer and the strongest evidence of a predictive effect has been shown for inactivating mutations in BRCA1 and BRCA2 [42], [43], [44], [45]. Preliminary evidence of an anti-tumour effect has been shown for mCRPC, with defects in several homologous recombination genes, including BRCA2, ATM and CHEK2 [7]. However, as ATM and CHEK2 mutations have not been described in other BRCA-associated cancers to date, here the evidence of a predictive effect is judged to be less, limited to prostate cancer and pre-clinical data [7], [40]. A balance is required in order to evaluate the treatment in the broadest patient group hypothesised to benefit, while accepting that where there is less evidence of a predictive effect, it may be appropriate to target a smaller treatment effect, thus requiring a larger randomised population. It should be considered whether to include a pre-planned subgroup analysis in the group(s) with the strongest evidence of a predictive effect; a step-down approach to analysis may be taken, as shown by other PARP trials [44], [46].

### Therapeutic Relevance

Only through pairing the biomarker-defined population with an effective therapeutic strategy can precision medicine approaches improve outcome. However, biomarker-treatment pairings may fail to translate to patient benefit due to an incomplete understanding of the biology of the biomarker or the interaction between the biomarker and the therapy [47]. Metastatic prostate cancer has been shown to be associated with multiple genetic aberrations, but in order to realise the therapeutic potential of this, it is necessary to distinguish mutations of significance. Ultimately, the highest level of evidence supporting an identified genetic alteration as an oncogenic driver and, therefore, a valuable therapeutic target, requires prospective evaluation within a clinical trial, including demonstration that biomarker-negative cohorts do not benefit from the targeted therapy [48]. Such evidence needs to be tumour site-specific due to the concept of epistasis, the gene–gene interactions proposed to explain the observed attenuated biological consequences of specific genetic aberrations according to tumour type. Examples of this include the differing impact of a BRAF V600E mutation, predictive of sensitivity to vemurafenib in melanoma and dabrafenib in non-small cell lung cancer, but not in the context of colorectal cancer. The latter is proposed to be due to the feedback of epidermal growth factor receptor, thus emphasising that driver classification requires contextual knowledge of other mutations present [49], [50].

One of the most well-described and seemingly perplexing challenges to the implementation of precision medicine approaches is intratumoural heterogeneity [51]. This can be considered as either spatial, the variation between different sites of disease, or temporal, the variation at different time points, for example pre- and post-treatment [52]. The key challenge to implementing precision medicine in metastatic prostate cancer is spatial heterogeneity, as this has the potential to introduce sampling bias [51]. Evidence from multi-regional sampling of cases of mCRPC obtained at rapid autopsy and sequential sampling shows metastatic spread to be polyclonal and polyphyletic, with evidence of metastatic-to-metastatic spread and both spatial and temporal heterogeneity [10]. This provides a powerful rationale to evaluate precision approaches in the first-line treatment of metastatic HNPC at the point closest to the sampling of the primary tumour. However, evidence of spatial heterogeneity will continue to motivate the investigation of alternative approaches to biomarker assessment, such as circulating tumour DNA. Such approaches are particularly relevant to this disease setting, given the predominance of bone metastatic involvement, which remains challenging to sample adequately to allow genetic analyses [53].

### Overlapping Biomarkers of Interest

In order for a trial platform such as STAMPEDE to evaluate multiple biomarker–therapeutic pairings, knowledge of the overlap between predictive biomarkers is required. This requires systematic profiling of a representative population; ongoing work being conducted as part of STAMPEDE aims to inform this. Genomic characterisation of clinical trial cohorts has been invaluable in other disease settings, such as those conducted as part of the S-CORT programme associated with FOCUS-4, which have informed biomarker prevalence, prognostic impact and overlap [54], [55]. If, for example, all cases of HRD prostate cancers overlap with a second biomarker of interest, then the feasibility of accruing to both comparisons would be dependent on the prevalence of both biomarkers or expanding accrual internationally. Greater understanding of the genetic profile of high-risk or metastatic prostate cancer will be crucial in identifying and designing future potential comparisons to be assessed in STAMPEDE.

## What Next for STAMPEDE?

In preparation for activating randomisation to the ‘rucaparib comparison’, a biomarker-screening pilot will start in late 2017. This will aim to establish the necessary processes to obtain rapid, prospective sequencing data prior to randomisation in order to determine eligibility. Following the pilot phase, biomarker screening will be activated in all participating centres when randomisation opens in early 2018. The development of this, the first biomarker-selected comparison, has highlighted the requirement for preliminary biomarker-focused research to inform the implementation of such approaches. The STAMPEDE protocol has included participant consent for the collection and analysis of archived tumour samples. As more outcome data become available there are a growing number of opportunities to support biomarker development through correlative analysis. Funded by Prostate Cancer UK, the STRATOSPHere consortium (STratification for RAtional Treatment-Oncomarker pairings of STAMPEDE Patients starting long-term Hormone treatment) aims to undertake a co-ordinated, multi-centre approach to generating preliminary data to accelerate the introduction of biomarker-selected comparisons with STAMPEDE.

One such future comparison in development aims to evaluate the addition of a checkpoint inhibitor in men with metastatic HNPC. Recognising that there are currently insufficient data to support the use of a predictive biomarker-enrichment strategy, an alternative approach to biomarker development is suggested. As part of the STRATOSPHere consortium, a parallel translational programme would aim to prospectively collect and characterise the randomised population. Then, if supported by external data, prospective enrichment may subsequently be implemented as part of the multi-stage design. Alternatively, it may be possible to power a subgroup analysis by biomarker status, based on the prevalence data acquired. Finally, in order to accelerate biomarker validation it will be important to establish the necessary infrastructure that allows clinical and molecular characterisation and endorses data sharing.

## Conclusion

The management of men with high-risk prostate cancer has lagged behind other tumour types where molecular characteristics routinely inform therapeutic choice. Knowledge gained from the genomic characterisation of CRPC cohorts, together with the evidence of the therapeutic relevance of DNA repair defects, provides the rationale to investigate a precision approach to treatment. Through further adaptation, STAMPEDE will evaluate the addition of a PARP inhibitor in this, the disease setting in which the greatest impact of outcome has been shown to date. The implementation of precision medicine approaches in low-frequency biomarker groups is challenging, but incorporating both biomarker-selected and -unselected randomisations within a single platform offers further efficiencies to the MAMS approach and ensures that the trial platform remains inclusive and attractive to patients, investigators and funders. Correlative translational analyses using STAMPEDE data offer unparalleled opportunity for biomarker development, which can continue to inform the trial design and generate the required preliminary data as laid out in the framework presented in this review. Through this latest adaption, we aim to ensure STAMPEDE remains innovative and continues to accelerate the acquisition of knowledge that will improve outcomes for men affected by high-risk prostate cancer.

## Statement of Financial Interest

C. Gilson has received funding to the MRC Clinical Trials Unit from Astellas and Clovis Oncology. S. Chowdhury discloses honoraria: GlaxoSmithKline, Novartis; consulting or advisory role: Clovis Oncology, Sanofi, Pfizer, Astellas Pharma, Janssen; speakers' bureau: Clovis Oncology, Sanofi, Pfizer, Astellas Pharma, Janssen; research funding: Sanofi, Johnson & Johnson. M. Sydes has received funding to the MRC Clinical Trials Unit from Astellas, Clovis Oncology, Janssen, Novartis, Pfizer and Sanofi-Aventis. M. Parmar directs the MRC Clinical Trials Unit at UCL, which receives educational grant funding and/or pharmaceuticals at reduced cost for the trial its conducts from the following companies: Astellas, Clovis Oncology, Janssen, Novartis, Pfizer, Sanofi-Aventis.
